# Young Broiler Feeding Kinematic Analysis as A Function of the Feed Type

**DOI:** 10.3390/ani9121149

**Published:** 2019-12-15

**Authors:** Diego Pereira Neves, Saman Abdanan Mehdizadeh, Mayara Rodrigues Santana, Marlon Sávio Amadori, Thomas Michael Banhazi, Irenilza de Alencar Nääs

**Affiliations:** 1 College of Agriculture Engineering, State University of Campinas, Campinas, São Paulo 13000-000, Brazil; diegopneves@gmail.com (D.P.N.); irenilza@gmail.com (I.d.A.N.); 2 Department of Mechanics of Biosystems Engineering, College of Agricultural Engineering and Rural Development, Agricultural Sciences and Natural Resources University of Khuzestan, Ahvaz, Khuzestan 6133613395, Iran; 3 Faculty of Agricultural Sciences, Federal University of Grande Dourados, Dourados-MS 79800-000, Brazil; m.nadafzadeh70@gmail.com (M.R.S.); shanesazan.m@gmail.com (M.S.A.); 4 Faculty of Health, Engineering and Science, University of Southern Queensland, Toowoomba Campus, Toowoomba 4350, QLD, Australia; Thomas.Banhazi@usq.edu.au; 5 PLF Agritech Pty. Ltd. Toowoomba 4350, QLD, Australia

**Keywords:** 2-D image analysis, beak gape, feed particle size, granulometry, motion analysis, poultry

## Abstract

**Simple Summary:**

The present study aims to compare the kinematic feeding variables of 3–4 days old broiler chickens using three different feed types: fine mash (F1), coarse mash (F2), and crumbled (F3); size was 476 µm, 638 µm, and 1243 µm, respectively. The head displacement and the maximum beak gape were automatically calculated by computational image analysis to find the feeding behavior of broilers. The results did not show strong correlations between birds’ weight, beak size (length and width), and the kinematic variables. The “catch-and-throw” movements in F1 (the smallest feed particle) generally occurred in the first mandibulation, while in F3 (the largest feed particle) occurred in the latest mandibulation. It can be suggested that the adoption of “catch-and-throw” in the latest mandibulations increases with larger particles.

**Abstract:**

Past publications describe the various impact of feeding behavior of broilers on productivity and physiology. However, very few publications have considered the impact of biomechanics associated with the feeding process in birds. The present study aims at comparing the kinematic variables of young broiler chicks (3–4 days old; 19 specimens) while feeding them with three different feed types, such as fine mash (F1), coarse mash (F2), and crumbled feed (F3). The feeding behavior of the birds was recorded using a high-speed camera. Frames sequences of each mandibulation were selected manually and classified according to the temporal order that occurred (first, second, third, or fourth, and further). The head displacement and the maximum beak gape were automatically calculated by image analysis. The results did not indicate strong correlations between birds’ weight, beak size (length and width), and the kinematic variables of feeding. The differences between the tested feed were found mostly in the first and second mandibulations, probably explained by the higher incidence of “catch-and-throw” movements in F3 (33%) and F1 (26%) than F2 (20%). The “catch-and-throw” movements in F1 (the smallest feed particle) mostly occurred in the first mandibulation, as in F3 (the largest feed particle) also occurred in the latest mandibulations. It might be suggested that the adoption of “catch-and-throw” in the latest mandibulations increases with larger particles. The kinematic variables in the latest mandibulations (from the third one on) seem to be similar for all feed types, which represent the swallowing phase. It might be inferred that the temporal sequence of the mandibulations should be essential to describe the kinematics of a feeding scene of broiler chickens, and the first and second mandibulations are potentially the key factors for the differences accounted by the diverse feed particle sizes.

## 1. Introduction

Although genetics is mainly responsible for the exceptional growth rates of modern poultry species, advances in feed and nutrition are the main drivers of cost-effectiveness in commercial broiler farms.

There is no actual chewing (mastication) in birds’ feeding behavior. The bird tongue is rigid, and the tactile sensibility is mostly perceived when the feed particles are touched and seized by the birds’ beak tip [1]. It has been suggested that broiler chickens can select different sizes of feed particles from the first week of life and respond to the stimulus of feed intake immediately after hatching [2]. The beak regulates the size of the feed that can be eaten, and the bird decides whether to accept or reject the particle [3]. Thus, the size of the feed particles, also known as granulometry and its shape, plays a vital role in the intake process of broiler chickens [4,5]. 

The feeding process of domestic chickens (*Gallus gallus domesticus*) is comparable to other birds, especially to pigeons (*Columbia livia*) [6,7], and can be described as: (1) The fixation phase, the head still stable above the target particle; (2) the approach phase, a uninterruptedly movement of the head towards food; (3) the grasping phase, when beak tip apprehends the particle; (4) the withdrawal phase, when the head is withdrawn in an upward motion; (5) the stationing phase, the particle is eventually repositioned in the beak tip; (6) the transporting phase, refers to the transport of the particle from the beak tip into the pharynx level; and (7) the swallowing phase, when occurs the final transportation of the seed into the esophagus. The feed particle size controls the amplitude of the beak opening, and the initial beak opening (grasping phase) is used to control the amplitude. Two forms of techniques have been described regarding the way that these birds handle the feed within the beak, the “catch-and-throw,” (positioning phase), and the “slide-and-glue” (transporting phase) movements. The first one occurs in the positioning phase and the second one in transporting the feed particles. It consists of repositioning the particles in the tip of the beak in the positioning phase, or similar movement could be used to transport the feed from the beak tip into the oral cavity. The second one occurs in the transporting phase and consists of the displacement of the tongue up to the tip of the beak to adhere to the feed with the aid of the sticky saliva and then proceed with the transport at the pharynx level [4].

Previous studies have addressed the impact of feed granulometry on consumption [8], the physiological responses to feeding [7,9,10], and the dynamic feeding process of chickens [5]. With the advancement of the high-speed cameras, it became possible to register the birds’ beak movements not previously recorded. The present research aimed at analyzing the feeding kinetics of young broiler chicks by accessing correlations between birds’ weight and beak size with feeding kinematic variables and compare birds’ head motion and maximum beak gape during mandibulations at three different feed types with a different granulometry of the ingested feed.

## 2. Material and Methods

A total of 19 male broiler chicks were individually recorded with a high-speed camera (Weinberger^®^, Visario 1500, Nürnberg, Germany) during feeding at the age of 3 and 4 days (d). From the first week of rearing, the broiler chicks learn to eat at the trough [2,3]. Thus, we prioritized the identification of the effectiveness of the eating process in young broilers. The high-speed camera was set at an acquisition rate of 250 fps (frames per second) at a resolution of 1536 × 1024 pixels, using a 50 mm F 1.4 lens (Nikon-F, Nikon, Tokio, Japan). The camera was placed at 1.0 m distance from the birds to allow framing their heads from a lateral-perpendicular orientation. A computer was connected to the camera for data acquisition, and a reflector LED spotlight (LED MR16, Philips, Amsterdam, The Netherlands) was the light source. 

**Figure 1:**
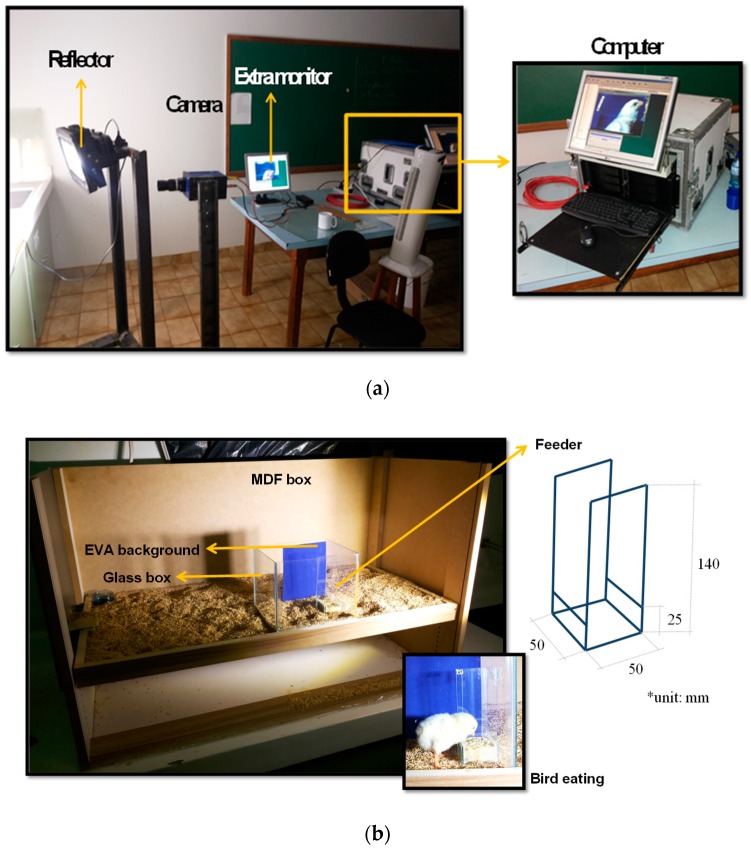
An overview image of the recording chamber (**a**), and the schematic and detail of chick eating at the glass trough inside the recording chamber (**b**).

The chicks were subjected to one-hour fasting before the recordings to stimulate the appetite [9]. Subsequently, the birds were randomly chosen from the experimental broiler house and transported to wooden boxes before they were selected to be placed in the recording chamber (Figure 1). The wooden boxes (100 cm length, 50 m width, 60 m height) had the same bedding material and bell-type drinker as the experimental house with water ad libitum, but still feed-restricted. Chicks were randomly selected and transferred from the wooden box and individually placed on the recording chamber (glass box with dimension 20 cm length, 15 cm width, 18 cm height) with a trough made of transparent glass, where the feeding scene was video recorded. Each bird was in the recording chamber with the trough for 20 min and returned to the box. The feed types were offered separately, and after the recordings, the birds were weighed, and morphometric traits of the beak (length and width) were measured with a digital caliper. Video calibration from pixels to SI dimensions was conducted using a ruler that was placed in the recording chamber.

**Figure 2:**
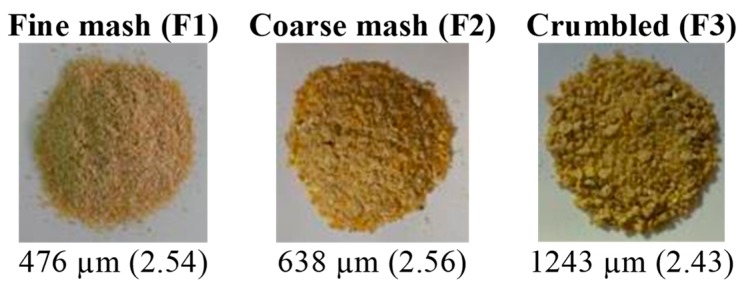
Images of the feed types used in the experiment with their respective geometric mean diameter and geometric standard deviation (between parentheses) [11].

Three different feed types were offered to the birds, fine mash (F1), coarse mash (F2), and crumbled (F3) [10]. The order of the presentation of the different feed types was randomized. The geometric mean diameter (GMD) and geometric standard deviation (GSD) of the feed particles (mash and crumble) were calculated [11] (Figure 2).

### 2.1. Description and Calculation of the Kinematic Variables

**Figure 3:**
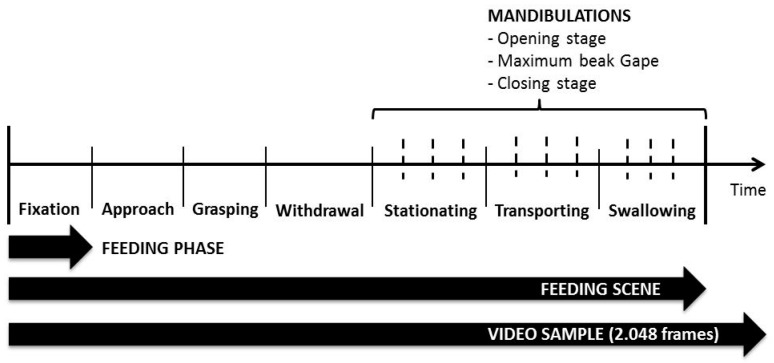
Representation of the chickens’ feeding scene over time. Fixation = the head still stable above the target particle; Approach = uninterruptedly movement of the head towards food; Grasping = beak tip apprehends the particle; Withdrawal = the head is withdrawn in an upward motion; Stationing = the particle is eventually repositioned in the beak tip; Transporting = the transport of the particle from the beak tip into the pharynx level; Swallowing = final transportation of the seed into the esophagus.

The mandibulation consisted of one cycle of opening and closing of the chick’s beak in the stationing, transport, and swallow phases (Figure 3). The beak opening started when beak began its aperture until its full opening (maximum beak gape) and then its closure. Ultimately, the beak was not entirely closed at the beginning of its aperture and the end of its closure. Frames sequences were classified per mandibulation and one single frame representing the maximum beak gape. Also, each mandibulation were classified in the temporal order that they occurred as first (1), second (2), third (3), fourth, and further on (4), starting right after the withdrawal phase until the last mandibulation before the birds start another feeding sequence in the fixation phase. The fourth classification comprehends the fourth and further mandibulations until the final swallowing. Also, the “catch-and-throw” movements adopted by the birds to repositioning and transport the feed were identified. 

The displacement of the birds’ head was analyzed automatically by tracking the bird’s eye position (2D) in the image, and then the total displacement during each mandibulation was calculated. The maximum beak gape in each mandibulation (i.e., the distance between upper and lower beak tips) was calculated automatically from the frame. A machine vision procedure [12] was used to carry out the calculations in Matlab^®^ software (MathWorks, Inc., Natick, MA, USA) without physical markers attached to the birds’ body. The technique consisted of the following steps: (1) initially eye detection was conducted (as a reference point) to determine the position of chick’s head; (2) then the head area was extracted to remove redundant background information (part of the picture that is of no interest). Such a procedure was followed by a (3) beak tips detection to analyze the maximum beak gape, and finally (4) the “removal” of the feed particle pixels was undertaken to inhibit possible errors during the detection of the beak tips in case of feed particles overlapping with the beak tips. A total of four kinematic variables were considered, (1) the maximum beak gape (mm); (2) the head displacement (mm); (3) the time of each mandibulations (ms) (calculated by the total number of frames); and (4) the head average speed (mm/s) during each mandibulation (calculated by the ratio between displacement and time).

### 2.2. Statistical Analysis

A total of 57 × 2048 frames from 19 specimens (three feed types) resulted in a total of 1729 mandibulations analyzed, being 576 for F1, 602 for F2, and 551 for F3. Minitab 15^®^ software (Minitab Inc., Pennsylvania, USA) was used to carry out the statistical analysis. Since the data were not normally distributed, non-parametrical tests were applied. At first, Spearman’s correlation test was used for the whole dataset considering the following variables, weight (g); beak length (mm); beak width (mm); head displacement (time); average speed (mm/s); and maximum beak gape (mm). After that, a correlation test was applied using the kinematic variables using the mandibulation order (1, 2, 3, and 4). The Mood’s median test was applied to verify the differences in the kinematic variables between feed types and mandibulation orders. The frequency of occurrence of “catch-and-throw” movements was identified, and the Chi-Square test was applied to establish the difference between feed types.

The experiments were carried out at the Federal University of Grande Dourados-UFGD, Brazil, and the trial was approved by the University Ethics Committee (Protocol number 030/2013-CEUA/UFGD).

## 3. Results

The Spearman’s correlation test (Table 1) did confirm some modest correlation between the birds’ weight and beak morphometric traits (length and width) with kinematic variables (head displacement, time, head average speed, and maximum beak gape). The strong positive correlation found between head average speed and head displacement was expected since the head average speed was calculated as the rate between head displacement and the time. However, it can be seen in Table 2 that the maximum beak gape presented moderate positive correlations with head displacement (0.635) and speed (0.503) in the first mandibulation, and the correlation coefficient decreased in the latest mandibulations along the time.

**Figure 4:**
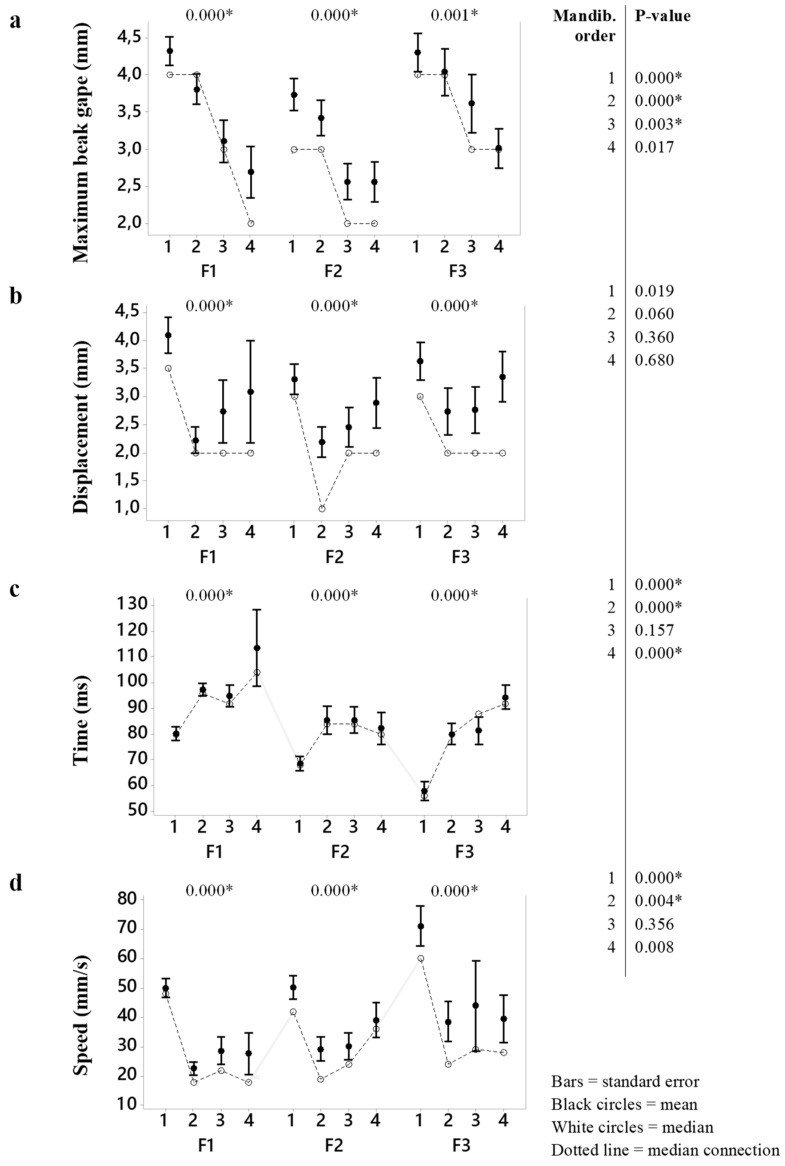
Kinematic variables per mandibulation order (1, 2, 3, and 4) and feed type (F1, F2, and F3). Significant differences (Mood’s median test; *p* < 0.05) between each mandibulation order by feed type are indicated in asterisk.

Table 3 presents the descriptive analysis of the kinematic variables per mandibulation order in each feed type. The Mood’s median test (Figure 4) indicated that all mandibulation orders at all feed types, and for all kinematic variables were different (*p* < 0.05). We found that when each mandibulation order per feed type was compared, they differed (*p* < 0.05), especially in the first and second mandibulations. The exception was for the head displacement at all mandibulations (indicated on the right side of Figure 3). 

The maximum beak gape (Figure 4a) was higher for F1 and F3 (4.0 mm) than F2 (3.0 mm) at the first and second mandibulations. The maximum beak aperture was observed in the largest and smallest granulometry. Probably the fine mash feed (F1) caused more difficulty to be ingested then the coarse mash (F2), especially at the first, second, and third mandibulations. This fact can be endorsed by the higher head displacement in the first mandibulation (Figure 4b). 

**Figure 5:**
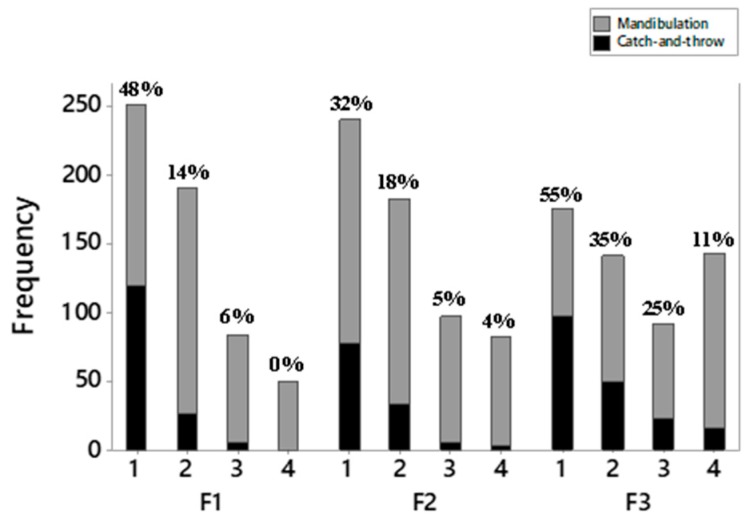
Percentage of “catch-and-throw” occurrences in each mandibulation order (1, 2, 3, and 4) and feed type.

The percentage of mandibulations that represent “catch-and-throw” movements was 33% for F3, 26% for F1, and 20% for F2 (*p* < 0.05). Figure 5 presents the frequency of “catch-and-throw” per mandibulation order in each feed type. The higher incidence of “catch-and-throw” in F3 is explained by the need to re-position of the feed within the beak, generally in the first and second mandibulations, and to aid the transportation into the oral cavity, mainly because of the higher incidence in the latest mandibulations. On the other hand, the “catch-and-throw” in F1 and F2 is applied to transport the feed into the oral cavity and not for repositioning. In F1, this is even more evident in the first mandibulation, and it was not detected in the latest mandibulations from the third one. The time expenditure associated with the latest mandibulations in F1 is more prolonged (*p* < 0.05) than other feed types (Figure 4c).

The head speed at F3 (crumbled feed-type) in the first mandibulation was higher than F1 and F2 (Figure 4d). The different speeds were not due to the range of the movement (head displacement), but because of the time in which they occurred. The F3 was the largest particle, so the total time expenditure of the mandibulations was shorter because the beak was not entirely closed at the beginning of its aperture; neither was it completely closed at the end of the mandibulation event. 

## 4. Discussion

The positive correlation between the maximum beak gape and head displacement was expected due to the cranial kinesis presented in all species of birds [6,13,14]. This feature is considered very important because of the facilitation of the highest elevation of the upper jaw, in which the quadrate bone plays a key role, as it can be seen as a central operating mechanism in the whole process [9,15,16,17]. Additionally, the cranial kinesis in domestic chickens is coupled, i.e., there is a relationship of dependence of both upper and lower jaws, although a certain degree of independence may occur [18]. Consequently, the chicken’s jaw is a unique structure that moves altogether; hence, this explains the positive correlations found between the maximum beak gape and head motion, mainly in the first mandibulation.

The results in the present study (Table 3) are similar to those described in previous studies on birds feeding behavior [6,7,19]. It has been suggested that the mechanism of the feeding of chickens is similar to that of pigeons [7]. Three feeding phases comprise mandibulations: stationing, transporting, and swallowing. Stationing consists in the repositioning of the feed particle in the beak tips by “catch-and-throw” movements, this technique is characterized by augmented movements of the head and beak aperture, but it can be skipped when the particle is adequately grasped. The transporting phase is the movement of the tongue up to the beak tip in order to glue the particle and transport into the oral cavity (so-called “slide-and-glue” movements) and is characterized by smaller movements of the head and beak aperture. The transporting phase can combine both “catch-and-throw” and “slide-and-glue” movements. The stationing phase compulsory presents “catch-and-throw” movements, but no “slide-and-glue,” while the transporting phase could present both types of movements. Lastly, the swallowing phase is the final transportation of the particle into the esophagus with movements of the pharynx, tongue, small beak openings, and head jerks, in which occur neither “catch-and-throw” nor “slide-and-glue.” According to these descriptions, it can be noticed in this study that the swallowing phase (from third mandibulations on) did not present significant differences for feed types.

On the other hand, differences occurred in the first and second mandibulations (stationing or transporting phases). The results found in this study suggest that the temporal sequence of the mandibulations should be essential to describe the kinematics of a feeding scene of broiler chickens, and the first and second mandibulations present the main differences between feed types. It has been suggested that these birds can adapt specific movement patterns depending on the type of feed particles, but such behaviors are related to limitations of the morphological structure [6].

According to Figure 3, it was expected that the maximum beak gape would be proportional to the feed size according to the findings of a previous study [7] when the authors compared the beak opening amplitude at six different sizes of spherical food pellet in pigeons. The granulometry of the tested feeds was 476 µm, 638 µm, and 1243 µm for F1, F2, and F3, respectively. Previous studies reported the impact of diet granulometry on feed consumption [8], and physiological responses to feeding [4,10,20] in broiler chickens, but little was found in current literature about the motion pattern of broiler chickens during feeding [5]. Regarding the particle sizes, the total time of the mandibulations, we found that the beak starts and finishes the mandibulation with its aperture consistent with the size of the particle, in agreement with the previous reports [7,19]. We found that the time spent in the latest mandibulations when particles are fine mash is more prolonged (*p* < 0.05) (Figure 4c), which means that chicks needed more time to swallow the feed. It can be argued that the adoption of “catch-and-throw” movements increases with larger particles and in further mandibulations until the final swallowing.

The muscles involved with feeding can be considered as biological motors that consume chemical energy and perform mechanical work. Thus, the functions of muscles influence the birds’ metabolism, in addition to other processes (thermoregulation), which likewise consumes oxygen (and ATP) and generates heat. The metabolic power of muscles is almost impossible to measure directly in vivo. However, the rate at which muscles can perform the mechanical work of feeding is limited by three mechanical variables: the stress or force that the muscles can produce, their range of motion, and their contraction frequency or velocity. The maximal values of these three variables are determined by the hierarchical structure of muscles from molecules and sarcomeres to whole muscle architecture [21,22]. Therefore, the kinematics of feeding considered in this study (maximum beak gape and head motion) relates at least indirectly to the metabolic cost of feeding. Consequently, it can be predicted that the chicks expended more energy when they adopted more “catch-and-throw” movements, which here was more prevalent in F1 and F3. On the other hand, the well-known physiological benefits of processed diets (e.g., crumble and pellet) that generally present larger feed particles could compensate for this energy expenditure, but this relationship was not testable in this study.

The method used in the present study to measure the beak opening was the distance between upper and lower beak tips. However, it is believed that the angle of beak aperture (considering beak tips and its vertex) could represent a more realistic condition for beak motion analysis. Small differences in beak shapes and sizes might imply similar beak apertures with different angles. Moreover, the beak motion analysis could present more correlations in older specimens, since it has been suggested that preferences for different feed sizes are more evident in older chickens because of the development of both digestive and buccal apparatus [8,23,24], which it should also be considered the improvement of jaws’ motor control during feed handling. Our results indicate that the size of the feed particle implies different kinematic of the feeding behavior of young broilers, which might lead to a difference in feed ingestion and consequent performance. Further studies should be carried out in all ages of broilers to verify if such feeding behavior continues as the birds grow until market weight.

## 5. Conclusions

No correlation was found between birds’ weight, beak size (length and width) with the studied kinematic variables. Moderate correlations were found for maximum beak gape with head displacement and speed, only in the first mandibulation right after the feed particle is grasped by the birds. Significant differences were found in the kinematic variables for all mandibulation orders at all feed types. Furthermore, differences between feed types were found mostly in the first and second mandibulations, probably explained by the higher incidence of “catch-and-throw” movements in F3 (33%) and F1 (26%) than in F2 (20%), but in F1 mostly in the first mandibulation. It can be suggested that the adoption of “catch-and-throw” movements increases with larger particles in further mandibulations until the final swallowing, as observed in the feed with the largest granulometry (F3). The latest mandibulations (from the third one on) seem to be similar for all feed types, which represent the swallowing phase. It can be suggested that the temporal sequence of the mandibulations should be essential to describe the kinematics of a feeding scene of broiler chickens, and the first and second mandibulations are potentially the key factors for the differences accounted by different feed particle sizes.

## Figures and Tables

**Table 1:** Spearman’s correlation tests between birds’ weight, beak length and width, and kinematic variables (head displacement, time, and average speed).

Items	Weight	Beak Length	Beak Width	Displacement	Time	Speed
Beak length (mm)	0.235 *					
Beak width (mm)	0.203 *	0.024				
Head displacement (mm)	−0.241 *	−0.141 *	−0.180 *			
Time (ms)	−0.310 *	−0.191 *	−0.082 *	0.154		
Head av. speed (mm/s)	−0.109 *	−0.063	−0.126 *	0.867 *	−0.294 *	
Max. beak gape(mm)	−0.211 *	−0.066	−0.139 *	0.403 *	0.142 *	0.337 *

* *p* < 0.05.

**Table 2:** Spearman’s correlation tests between kinematic variables (head displacement, time, average speed, and maximum beak gape) by mandibulation order.

Items	Mandibulation Order	Displacement	Time	Speed
Time (ms)	1	0.444 *		
	2	0.251 *		
	3	0.142		
	4	0.189 *		
Head av. speed (mm/s)	1	0.817 *	−0.088 *	
	2	0.878 *	−0.159 *	
	3	0.897 *	−0.237 *	
	4	0.861 *	−0.263 *	
Max. beak gape (mm)	1	0.635 *	0.349 *	0.503 *
	2	0.409 *	0.376 *	0.264
	3	0.154	0.131	0.119
	4	0.137	0.118	0.114

* *p* < 0.05.

**Table 3:** Descriptive analysis (mean, standard error, and median) of the kinematic variables for each for mandibulations orders by feed type.

Variable	Mandibulation Order	F1		F2		F3	
		Mean ± SE	Med	Mean ± SE	Med	Mean ± SE	Med
Max. beak gape (mm)	1	4.3 ± 0.10	4	3.7 ± 0.11	3	4.3 ± 0.13	4
	2	3.8 ± 0.10	4	3.4 ± 0.12	3	4.0 ± 0.16	4
	3	3.1 ± 0.14	3	2.6 ± 0.12	2	3.6 ± 0.20	3
	4	2.7 ± 0.17	2	2.5 ± 0.13	2	3.0 ± 0.16	3
Head displacement (mm)	1	3.1 ± 0.13	3.5	2.4 ± 0.11	3	2.4 ± 0.13	3
	2	1.2 ± 0.08	2	1.1 ± 0.08	1	1.5 ± 0.14	2
	3	1.8 ± 0.23	2	1.3 ± 0.12	2	1.6 ± 0.17	2
	4	1.8 ± 0.37	2	1.4 ± 0.14	2	1.7 ± 0.14	2
Time (ms)	1	80.3 ± 1.4	80	68.7 ± 1.4	68	57.9 ± 1.8	56
	2	97.4 ± 1.2	96	85.4 ± 2.8	84	80.1 ± 2.0	80
	3	95.0 ± 2.2	92	85.6 ± 2.6	84	81.4 ± 2.7	88
	4	113 ± 7.5	104	82.3 ± 3.2	80	94.4 ± 2.4	92
Head av. speed (mm/s)	1	1.0 ± 0.05	48	0.8 ± 0.05	42	1.3 ± 0.08	60
	2	1.0 ± 0.07	18	1.0 ± 0.08	19	1.2 ± 0.10	24
	3	0.9 ± 0.10	22	1.0 ± 0.12	24	1.1 ± 0.10	29
	4	1.2 ± 0.22	18	1.4 ± 0.15	36	1.5 ± 0.15	28

SE = standard error; Med = median; F1 = fine mash; F2 = coarse mash; F3 = crumbled.
